# The timing of reproduction is responding plastically, not genetically, to climate change in yellow‐bellied marmots (*Marmota flaviventer*)

**DOI:** 10.1002/ece3.10780

**Published:** 2023-12-06

**Authors:** Sophia St. Lawrence, Daniel T. Blumstein, Julien G. A. Martin

**Affiliations:** ^1^ Department of Biology University of Ottawa Ottawa Ontario Canada; ^2^ Department of Ecology and Evolutionary Biology University of California Los Angeles California USA; ^3^ The Rocky Mountain Biological Laboratory Crested Butte Colorado USA

**Keywords:** climate change, *Marmota flaviventer*, microevolution, phenotypic plasticity, quantitative genetics, reproduction

## Abstract

With global climates changing rapidly, animals must adapt to new environmental conditions with altered weather and phenology. The key to adapting to these new conditions is adjusting the timing of reproduction to maximize fitness. Using a long‐term dataset on a wild population of yellow‐bellied marmots (*Marmota flaviventer*) at the Rocky Mountain Biological Laboratory (RMBL), we investigated how the timing of reproduction changed with changing spring conditions over the past 50 years. Marmots are hibernators with a 4‐month active season. It is thus crucial to reproduce early enough in the season to have time to prepare for hibernation, but not too early, as snow cover prevents access to food. Importantly, climate change in this area has, on average, increased spring temperatures by 5°C and decreased spring snowpack by 50 cm over the past 50 years. We evaluated how female marmots adjust the timing of their reproduction in response to changing conditions and estimated the importance of both microevolution and plasticity in the variation in this timing. We showed that, within a year, the timing of reproduction is not as tightly linked to the date a female emerges from hibernation as previously thought. We reported a positive effect of spring snowpack but not of spring temperature on the timing of reproduction. We found inter‐individual variation in the timing of reproduction, including low heritability, but not in its response to changing spring conditions. There was directional selection for earlier reproduction since it increased the number and proportion of pups surviving their first winter. Taken together, the timing of marmot reproduction might evolve via natural selection; however, plastic changes will also be extremely important. Further, future studies on marmots should not operate under the assumption that females reproduce immediately following their emergence.

## Graphical abstract

Yellow‐bellied marmots reproduction timing is strongly linked to environmental conditions in the spring. If reproducing early is beneficial for pup survival, we found no genetic variation in the plasticity to environmental conditions, with all females responding in the same way to climate variation despite moderate genetic variation in the timing of reproduction.

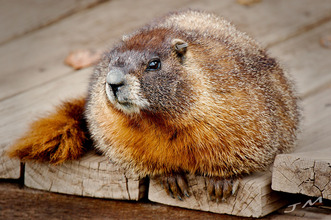

## 1 INTRODUCTION

Life history traits are those that impact the fitness of an individual through survival and/or reproduction (Braendle et al., 2011). The seasonal timing of these traits is heavily dependent on environmental conditions (Brommer, 2000; Bronson, 2009). These environmental conditions can vary inter‐annually (Bright Ross et al., 2020) and seasonally, in both the mean value of the environment and in the timing of important events (e.g., when food becomes available; Nussey et al., 2005). Animals must react to these yearly and seasonal variations by adjusting the timing of their life history traits to coincide with the environmental conditions that will maximize survival and/or reproduction. For example, timing egg laying dates so that food availability is at its highest at the peak of offspring food demand (Nussey et al., 2005), timing changes in coat colouration to match seasonal changes in the environment and thus avoid predation (Mills et al., 2013), and timing emergence from hibernation to emerge late enough that food resources are not covered by snow but early enough to maximize the length of the active season (Edic et al., 2020). However, when these environmental conditions occur, may be impacted by climate change (Gienapp et al., 2014; Mills et al., 2013; Nussey et al., 2005; Parmesan, 2006). For example, changes in the timing of food availability (Nussey et al., 2005) and in average season lengths have been documented (Cordes et al., 2020). These changes can lead to mismatched timing between animal behaviours and optimal environmental conditions if animals are not able to adjust their timing adequately. Consequently, fitness can be negatively impacted, and indeed, declines in both reproductive success and survival have been reported (Bailey et al., 2022; Cordes et al., 2020; Gienapp et al., 2014; Nussey et al., 2005). Animals can alter the timing of their life history traits to coincide with the changed timing of environmental conditions through phenotypic plasticity and/or microevolution (Boutin & Lane, 2014; Gienapp & Brommer, 2014; Visser, 2008).

Phenotypic plasticity occurs when a phenotype changes in response to a changing environmental condition (Nussey et al., 2007). This can be measured in a wild population by observing how a trait that is expressed multiple times during an individual's life changes in response to changes in climate (Nussey et al., 2007). Phenotypic plasticity is an important mechanism by which individuals respond to their environment, as it allows for a fast change in the phenotype that can accurately track sudden changes in environmental conditions (Charmantier et al., 2008; Merilä & Hendry, 2014). Plasticity therefore also provides a potentially important solution in terms of climate change response because, while the trend in climatic changes is expected to be directional (Boutin & Lane, 2014), variability is expected to increase (Childs et al., 2010).

However, the capacity of phenotypic plasticity to respond to these global environmental changes may be limited over the long term (Boutin & Lane, 2014; Merilä & Hendry, 2014). Certain studies have shown that to fully adapt to changes in climatic conditions, populations will need to undergo microevolutionary changes, as phenotypic plasticity will not be enough on its own (Mills et al., 2013; Phillimore et al., 2010). These microevolutionary changes can occur in plasticity or in the mean of the trait if there is phenotypic variation, heritability, and selection. However, while microevolution may offer a long‐term solution to responding to climate change, it may not be fast enough to ensure species persistence (Boutin & Lane, 2014; Gienapp et al., 2007; Radchuk et al., 2019). Microevolution is a relatively slow and not easily adjustable process in comparison to phenotypic plasticity; this may prove problematic as a response to climate change, which can occur quickly and vary inter annually (Charmantier et al., 2008; Radchuk et al., 2019).

Despite the extensive background research on these topics, studies examining the relative contributions of phenotypic plasticity and/or microevolution in the response of a wild population to climate change were rare (Boutin & Lane, 2014; Merilä & Hendry, 2014; Nussey et al., 2007) but are rapidly increasing in number (Bailey et al., 2022; de Villemereuil et al., 2020; Radchuk et al., 2019). Studies of this nature require long‐term data, a known pedigree, a sizeable population, and a study site that is impacted by climate change (Boutin & Lane, 2014; Gienapp & Brommer, 2014; Nussey et al., 2007). One example of a study system that meets these requirements are the yellow‐bellied marmots (*Marmota flaviventer*) of the Rocky Mountain Biological Laboratory (RMBL) in Colorado, USA. Since 1962, this study system has generated annual data on individual marmots. Maternity has been assigned behaviourally since the study's beginning, and paternity assignment began in 2000. For the past 50 years, there has been a steady increase in mean temperatures and a decrease in mean snowpack, with one of the fastest reported changes in spring climate. Specifically, there has been an increase of 5°C in average spring temperatures and a decrease of 50 cm in average spring snowpack over the past 50 years (Figure S1).

Coupled with these climatic changes are changes in the marmots' life history: adult emergence date from hibernation has advanced (Edic et al., 2020), pups are being weaned earlier (Ozgul et al., 2010), and overwinter survival is decreasing while summer survival is increasing (Cordes et al., 2020). Indeed, a marmot's life history is heavily constrained by climate (Cordes et al., 2020). During the short 4‐month growing season, marmots must gain as much weight as possible to survive hibernation (Ozgul et al., 2010). This mass gain will be influenced by their date of birth in their first year and their date of reproduction in subsequent years. Individuals that are born later are less likely to survive overwinter than those born earlier in the season (Monclús et al., 2014). Similarly, if a female is investing energy and resources into lactating late into the season, she may also have a harder time surviving overwinter than those that invest earlier (Andersen et al., 1976). We might assume that marmots should emerge and reproduce earlier to increase the length of this crucial growing season. However, emerging and reproducing too early also poses problems. If there is still snow on the ground covering food resources when marmots emerge, they must draw on depleted energy stores for longer (Cordes et al., 2020). This could potentially lead to starvation, decrease the number of pups in a litter, or cause marmots to forgo reproduction altogether (Inouye et al., 2000). Nevertheless, there has been a documented advance in the emergence date from hibernation (Edic et al., 2020).

Since marmots are thought to reproduce immediately following emergence, we expected the timing of reproduction, pup emergence date, and adult emergence date to be strongly linked and to follow a similar pattern. However, how the timing of reproduction varies from year to year and the impact of climate change on the timing of reproduction remain unknown. Therefore, we were interested in examining whether the timing of reproduction is changing in response to changes in average spring temperatures and average spring snowpack. Given that these changes can occur through microevolution and/or phenotypic plasticity, we further investigated the relative contributions of each by examining whether the trait is heritable, whether there is selection on the timing of reproduction in response to climate change, and whether there is phenotypic plasticity in the trait in response to changes in both average spring temperature and snowpack. As reproduction is expected to occur immediately following adult emergence, we expected the results for the timing of reproduction and adult emergence to be similar. Therefore, following Edic et al. (2020), we expected there to be low but estimatable heritability for the trait, strong plasticity, and an impact of both spring temperature and snowpack. In addition, since individuals often differ in their responses to environmental conditions, we expected IxE in plasticity. Finally, we expected linear selection on the timing of reproduction since pups born too late would have a shorter period to grow.

## 2 METHODS

### 2.1 Study species and data collection

Yellow‐bellied marmots are large (adult females weighing on average 2.5 kg and adult males weighing on average 3 kg; Armitage, 2014) hibernating rodents living up to 15 years. They have a 4‐month active season, from late‐April/early‐May to late‐September, during which they need to reproduce and accumulate fat reserves to survive the 8 months of hibernation (Armitage, 2014). A population of yellow‐bellied marmots has been studied at the Rocky Mountain Biological Laboratory (RMBL) in Gothic, CO, USA since 1962.

Marmots were live trapped in Tomahawk traps regularly during the active season. When they were caught, data on their weight, sex, and reproductive status were collected. Upon first trapping, marmots were assigned a unique identifier and given a permanent ear tag for identification. For observations at a distance, Nyanzol‐D, a semi‐permanent dye, was applied in a unique pattern to each marmot. Since 2000, parentage has been determined using genetic assignment (for detailed methodology on genetic assignment, see Blumstein et al., 2010). Prior to this, maternal identity could be reliably determined via behavioural observations, while paternal identity remained unknown since males do not contribute to parental care. Daily climate data have been collected by an on‐site weather station since 1975. Data collected included daily minimum and maximum temperatures, daily precipitation, and the depth of the snowpack. Mass on June 1st and August 15th were estimated for each individual every year using the best linear unbiased predictors from age‐ and sex‐specific linear mixed models (for detailed methods, see Kroeger et al., 2018). For pups, mass was estimated for the emergence date and not June 1st. Age was calculated using the birth year and the year of capture. Since 83% of females are captured for the first time when they are juveniles, they are of known age. The study is divided into an up‐valley and a down‐valley that differ in elevation by 165 m (Ozgul et al., 2010), resulting in a delay in the phenology of the up‐valley by about 2 weeks compared to the down‐valley (Monclús et al., 2014). Within the up‐valley and down‐valley sites, social groupings of matrilines with at least one reproducing male form main colonies, whereas satellite colonies are formed by lone or small groups of marmots (St. Lawrence, Dumas, et al., 2022; Svendsen, 1974). For our analysis, we restricted our dataset to include only the six main colonies, as they have over 80% of the individuals that are observed each year compared to the 20 satellite colonies. Observation effort is much higher at main colonies, and thus the accurate emergence date of adult females is available only at main colonies. The emergence date of females was estimated as the day they were first observed, measured in the days of year.

To estimate the timing of reproduction, we used the date pups first emerge from their burrow after being weaned as a proxy, measured as day of year (number of day since January 1). Since the length of gestation and lactation are considered fixed in the marmots, with 30 days spent gestating and 25 days spent lactating (Armitage, 2014), and pups emerge immediately following weaning (Monclús et al., 2014), this is an excellent proxy for the timing of reproduction. Given that adult emergence in the spring is related to average spring daily mean temperature and average spring snowpack, we focused on these two environmental variables for our analysis of pup emergence date. Since seasonal averages of environmental variables can be estimated between any two arbitrary timepoints (Bailey & van de Pol, 2016), we used a statistical approach to determine which phenological window of these two variables had the greatest association with pup emergence date. This was done using the statistical approach built into the R package climwin (Bailey & van de Pol, 2016; van de Pol et al., 2016). This package allows the fitting of multiple models with different phenological windows used to estimate environmental averages and determine, using AIC‐based model comparison, which window has the strongest relationship with the biological variable of interest.

### 2.2 Statistical analyses

All statistical analyses were conducted in R v.4.0.3 (R Core Team, 2020) using packages climwin v.1.2.3 (Bailey & van de Pol, 2016; van de Pol et al., 2016), lme4 v.1.1.26 (Bates et al., 2015), asreml‐R v.4.1 (Butler, 2021), ggplot2 v.3.3.3 (Wickham, 2016), tidyverse v.1.3.1 (Wickham et al., 2019), nadiv v.2.17.1 (Wolak, 2012), lubridate v.1.7.10 (Grolemund & Wickham, 2011), gridExtra v.2.3 (Auguie, 2017), ggeffects v.1.1.2 (Lüdecke, 2018), lmerTest v.3.1.3 (Kuznetsova et al., 2017), and car 3.0.10 (Fox & Weisberg, 2019). For all models, all continuous variables fit as fixed effects and were scaled to have a variance of one and a mean of zero.

One of the key assumptions in a marmot's life history is that they reproduce immediately following their emergence. We wanted to investigate this assumption by estimating the relationship between female emergence from hibernation and pup emergence date from weaning in a given year. To do so, we used a restricted dataset from the years 2003 to 2017, where we had all female emergence dates from hibernation and pup emergence from weaning in each year. We then ran a linear mixed model with the package lme4 (Bates et al., 2015) using pup emergence date as our response variable. We used female emergence date and litter size as fixed effects. Year and female identity were fitted as random effects. Since we were expecting a 1:1 ratio between pup and female emergence date, we also used a *t*‐test to compare the slope of the female emergence date to 1.

To determine during which phenological window temperature and snowpack had the strongest association with pup emergence date, we used the R package climwin (Bailey & van de Pol, 2016; van de Pol et al., 2016). For both environmental variables, we fit a linear mixed model in climwin with pup emergence date as our response variable, and our independent variables were time (number of years since the start of the study) and either daily average temperature or snowpack. We also included the year of measurement and female identity as random effects to take into account repeated measurements in the data. Our specified reference day was June 1st, and the starting date of the window varied from June 1st to November 13th (200 days before June 1st) the previous year. We used an absolute window and allowed any length of window from 1 to 200 days. In addition, we ran a randomization approach with 500 permutations of the data to verify that spurious environmental effects were not detected.

To investigate whether climate change was impacting the timing of reproduction in the yellow‐bellied marmot, we fitted a univariate animal model of pup emergence date using the asreml‐R package (Butler, 2021). Fixed effects included the mother's age, valley (up‐ or down‐valley), average spring snowpack, average spring temperature, litter size, and the mother's mass in June. Random effects were the year, permanent environment (see Kruuk & Hadfield, 2007), additive genetic, and colony effects. Year was added to control for inter‐annual variation in conditions experienced. The permanent environment effect was added to control for any inter‐individual variation in pup emergence date not due to genetic effects. Additive genetic effects were added to estimate the amount of variation in the phenotype associated with additive genetic variation. A colony was added to control for potential micro‐environmental differences between them. The significance of fixed effects was assessed using a Wald *F* test with a Kenward‐Rogers approximation for the denominator degrees of freedom. For random effects, significance was determined using a log‐likelihood ratio test. Starting from our full model, random effects were dropped one at a time, and the log‐likelihood ratios of each model were compared. Summary statistics of the pruned pedigree used in the animal models can be found in Table S2.

To investigate inter‐individual variation in the plasticity of the timing of reproduction, we modified the previous model by adding random slopes for individuals for average spring snowpack and temperature in two separate models and also removing the additive genetic effect using asreml‐R (Butler, 2021). We tested the significance of the random slope terms and the random intercept by comparing the log‐likelihood of the models with and without each of these effects. Given that no variation in random slopes was detected, models including additive genetic random slopes were not fitted.

We estimated the existence of selection (directional and/or stabilizing) on the pup emergence date. To do this, we ran generalized linear mixed models with three different fitness proxies in lme4 (Bates et al., 2015). For each litter, we used the total number of pups surviving to 1‐year‐old, the proportion of pups surviving to 1‐year‐old (weighted to account for litter size), and the total number of pups. We used the same model structure for the three models. Fixed effects were the linear and quadratic orthogonal polynomials of pup emergence date, mother's age, mother's mass in June, average spring snowpack, average spring temperature, and valley. Random effects were female identity, colony, and year. We used a Poisson distribution for the number of pups surviving to 1‐year‐old and the number of pups in the litter. We used a binomial distribution for the proportion of pups surviving to 1‐year‐old.

## 3 RESULTS

### 3.1 Relationship between female emergence date from hibernation and pup emergence date

**FIGURE 1:**
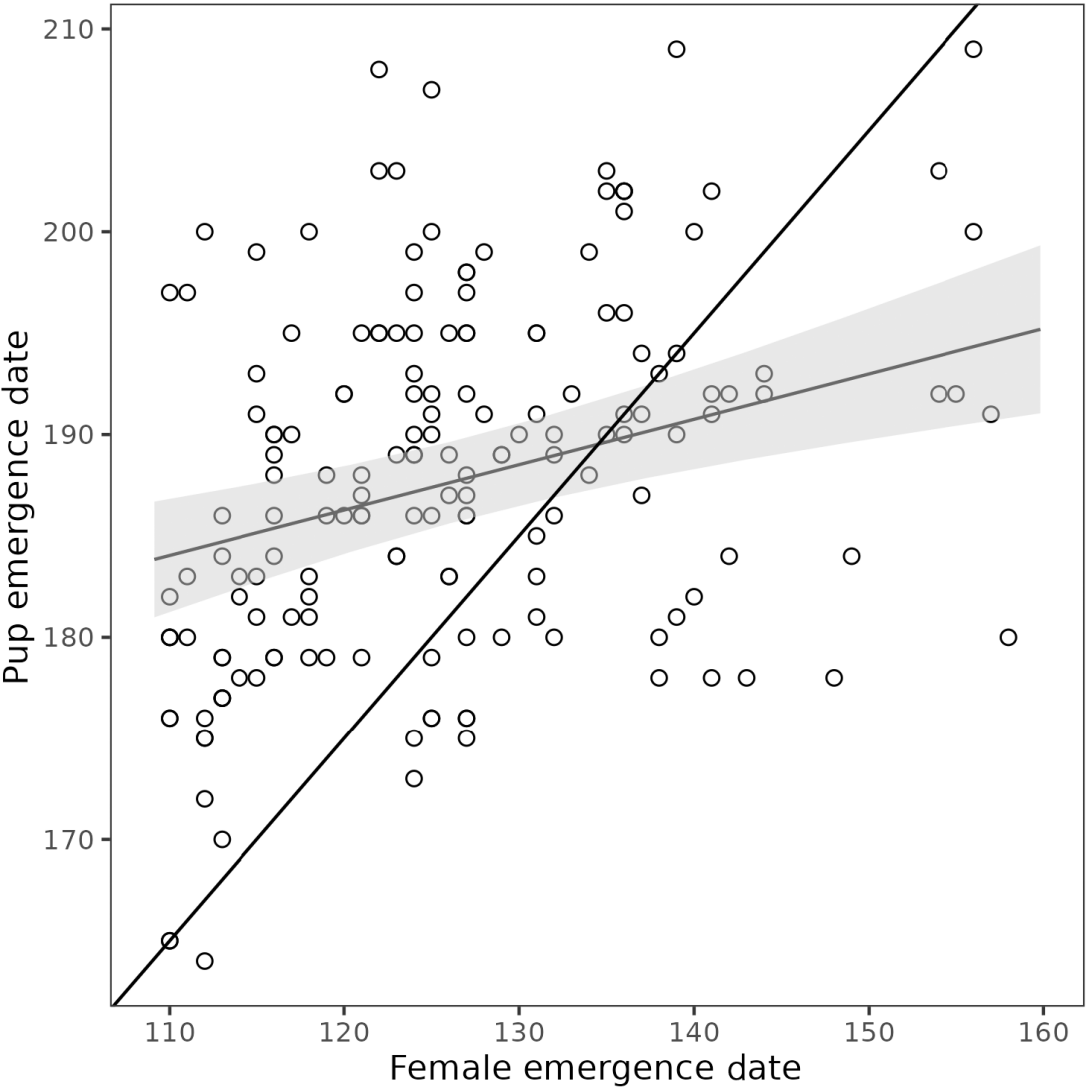
Relationship between female emergence date and pup emergence date. Thin black line represents the predicted slope if females were reproducing immediately after emerging. Darker black line represents the observed relationship between pup emergence date and female emergence date (number of females = 88, number of litters = 171).

Female emergence date was a significant predictor of pup emergence date, with females that emerged later producing pups that emerged later (Estimate ± SE = 0.282 ± 0.071; Table S1, Figure 1). However, the slope of the relationship between pups' and females' emergence dates was also significantly different from 1 (*t*_134.1_ = −10.11, *p* < .001). Some females are having their pups emerge earlier than would be expected based on their emergence date, while the majority are having their pups emerge later than would be expected (Figure 1), indicating that some females reproduced before emerging and most delayed reproduction after emergence (Figure 1). We also found that for a given female emergence date, larger litters emerged on average earlier than smaller ones (−0.082 ± 0.035, *t*_166.827_ = −2.335, *p* = .021).

### 3.2 Determinants of pup emergence date

**FIGURE 2:**
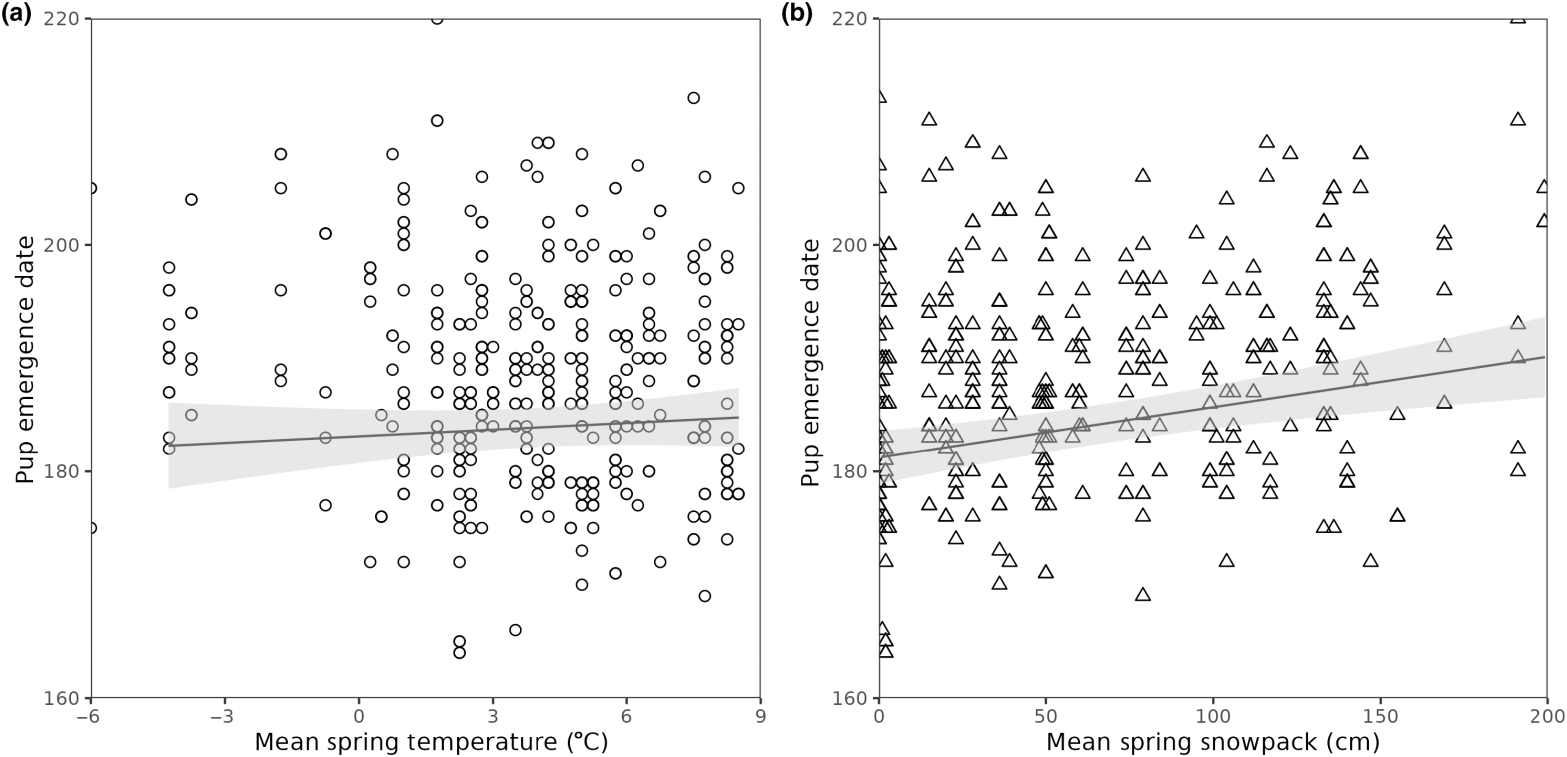
Relationships between climate variables (a—mean spring temperature [°C]; b—mean spring snowpack [cm]) and pup emergence date (number of females = 192; number of litters = 461).

**FIGURE 3:**
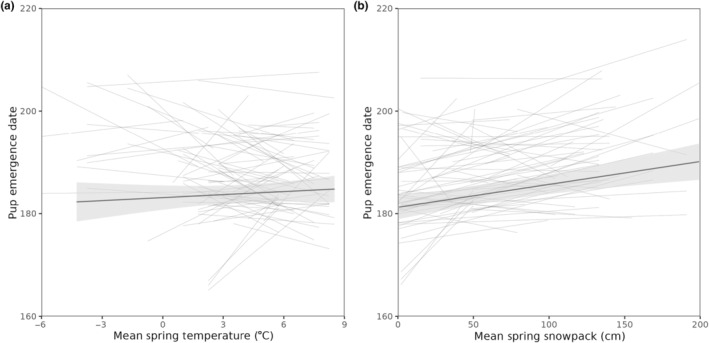
Relationships between climate variables (a—mean spring temperature [°C]; b—mean spring snowpack [cm]) and pup emergence date. The black, bold line represents the average individual response. Each thin grey line represents a unique female, with the length of the line showing the range of weather conditions measured for that female. The plot has been filtered to include only those females with 3 or more litters to enable clearer visualization. (number of females = 73; number of litters = 303).

The window of mean temperature and mean snowpack that had the strongest association with the timing of reproduction was between mid‐April and early‐May (Figure S2) with the best window opening on April 15th and 25th and closing on May 1st and 3rd for snow and temperature, respectively. Given that the windows corresponded to previously reported patterns (Armitage, 2014), the mean spring temperature and snowpack were defined as before between April 15th and May 5th. Average spring snowpack was positively related to pup emergence date, but average spring temperature was not (Table 1, Figure 2). Pup emergence date was also affected by valley, with pups emerging later in the up‐valley compared to the down‐valley (Table 1). Heritability of pup emergence date was low, approximately 8%, and not significantly different from zero (Table 2). We also report significant year and permanent‐environment variance in the date of pup emergence (Table 2). There was no statistically significant inter‐individual variation in the degree of plasticity nor covariation between the intercept and the slope for either spring snowpack (Slope Variance ± SE = 0.39 ± 1.68, Intercept/Slope Covariance ± SE = 1.07 ± 1.69, LRT_2_ = 0.40, *p*‐value = 0.82) or spring temperature (Slope Variance ± SE = 0.02 ± 1.45, Intercept/Slope Covariance ± SE = 0.27 ± 1.69, LRT_2_ = 0.01, *p*‐value = 1) (Figure 3).

**TABLE 1:** Fixed effect estimates from the model of pup emergence date (number of females = 184; number of litters = 425, mean number of observations per female = 2.3, range of observations per female = 1–9).

	Estimate	Standard error	*F* test	df	*p*‐Value
Intercept	−0.364	0.138	1.026	1, 35.4	.318
**Average spring snowpack**	**0.253**	**0.068**	**13.96**	**1, 42.8**	**.001**
Average spring temperature	0.056	0.067	0.7	1, 37.2	.408
Female's age	0.015	0.048	0.102	1, 406.8	.749
Female's mass in June	−0.084	0.062	1.834	1, 364.3	.176
Litter size	−0.025	0.021	1.378	1, 411.4	.241
**Valley [up]**	**0.962**	**0.124**	**59.95**	**1, 24.6**	**<.001**

*Note*: df is the numerator and denominator degrees of freedom for the *F* statistic. Reference category is down‐valley for valley. Pup emergence date was scaled to have a variance of one and a mean of zero. Effects with *p* values below .05 were highlighted in bold.

**TABLE 2:** Variance components and ratios for colony, year, additive genetic, and permanent environment from the univariate animal model analysing the association of changing average spring snowpack and temperature with pup emergence date (number of females = 184; number of litters = 425).

Variables	Variance component (estimate ± SE)	Variance ratio (estimate ± SE)	LRT	*p*‐Value
Colony	0.000 ± NA	0.000 ± 0.000	0.000	1
**Year**	**0.083 ± 0.034**	**0.120 ± 0.045**	**18.413**	**<.001**
Additive genetic	0.054 ± 0.061	0.072 ± 0.085	0.757	.384
Permanent environment	**0.169 ± 0.065**	**0.244 ± 0.091**	**7.772**	**.005**
Residual variance	0.390 ± 0.037			

*Note*: Effects with *p* values below .05 were highlighted in bold.

### 3.3 Selection analysis

**FIGURE 4:**
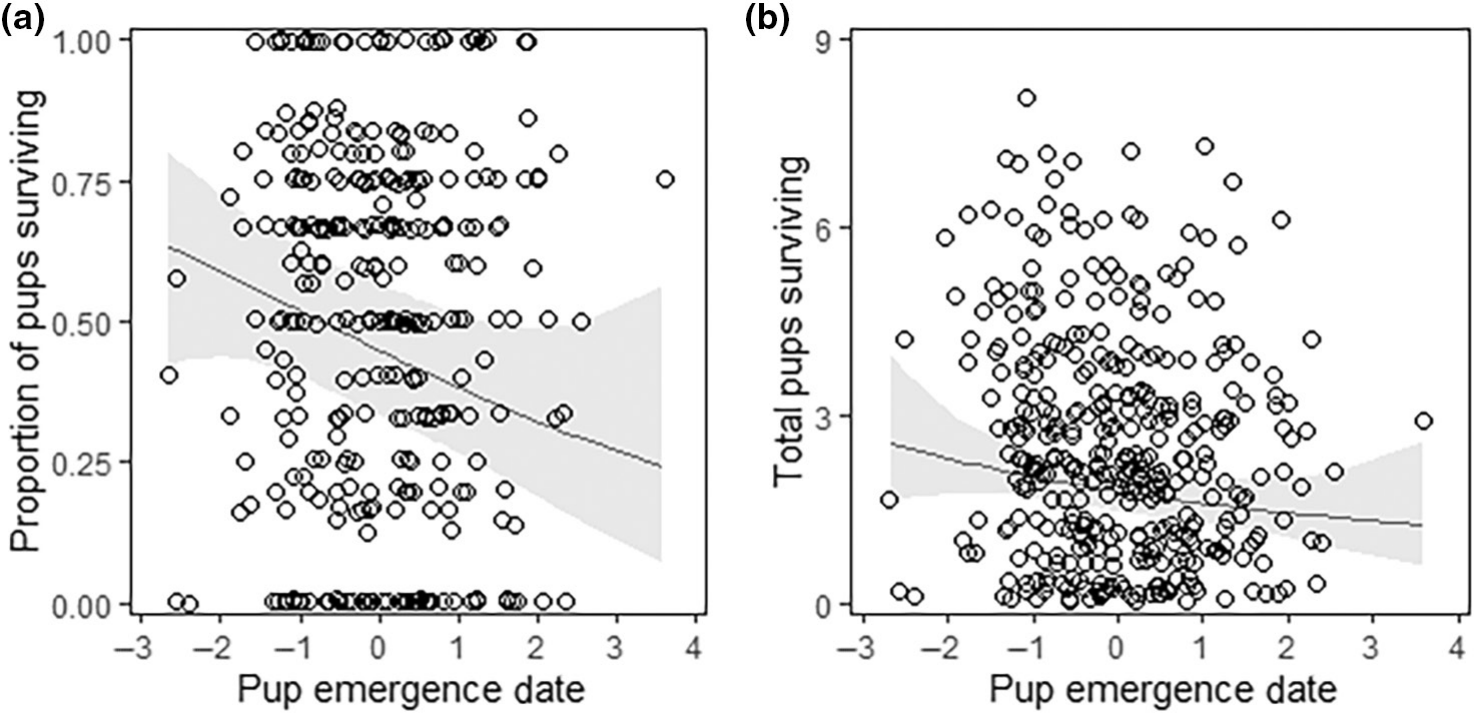
Relationships between pup emergence date and fitness proxies. (a) Output of our selection model, examining the relationship between pup emergence date and the weighted proportion of pups surviving their first winter. (b) Output of our selection model examining the association of pup emergence date with the total number of pups surviving their first winter. The black line represents the predictions from the models. The grey shading is the associated confidence intervals. Data points have been jittered to enable clearer visualization (number of females = 176; number of litters = 417).

For litter size, we found only a positive effect of the mother's mass in June and no effect of pup emergence date (neither linear nor quadratic) (Table 3). For the proportion of pups in a litter surviving to 1‐year‐old, we found only a negative linear effect of pup emergence date (Table 3, Figure 4a). For the total number of pups in a litter surviving to 1‐year‐old, we found a positive effect of maternal mass in June and a negative linear effect of pup emergence date (Table 3, Figure 4b). In addition, we found an effect of the valley, with more pups surviving to 1‐year‐old in the up‐valley compared to the down valley (Table 3).

**TABLE 3:** Estimates from generalized linear mixed model (GLMM) (number of females = 176; number of litters = 417) determining the association of pup emergence date with the weighted proportion of pups surviving their first winter (binomial distribution), the total number of pups surviving their first winter (Poisson distribution), and litter size (Poisson distribution).

	Weighted proportion				Total pups				Litter size			
	Estimate	Standard error	*Z*‐value	*p*‐Value	Estimate	Standard error	*Z*‐value	*p*‐Value	Estimate	Standard error	*Z*‐value	*p*‐Value
Intercept	−0.209	0.239	−0.874	.382	**0.583**	**0.093**	**6.297**	**<.001**	**1.510**	**0.055**	**27.693**	**<.001**
Female's mass in June	0.149	0.109	1.367	.172	**0.189**	**0.059**	**3.208**	**.001**	**0.134**	**0.035**	**3.803**	**<.001**
Female's age	−0.034	0.086	−0.400	.689	−0.020	0.046	−0.440	.660	−0.047	0.030	−1.578	.114
Valley_uv	0.528	0.294	1.793	.073	**0.238**	**0.104**	**2.281**	**.023**	−0.022	0.080	−0.277	.782
Average spring snowpack (cm)	0.190	0.173	1.097	.272	0.112	0.081	1.381	.167	0.039	0.038	1.029	.304
Average spring temperature (°C)	−0.072	0.172	−0.419	.675	0.020	0.079	0.258	.796	0.030	0.035	0.838	.402
Pup emergence date	**−5.580**	**1.846**	**−3.022**	**.003**	**−2.402**	**0.979**	**−2.454**	**.014**	−0.799	0.619	−1.292	.196
Pup emergence date^2^	0.075	1.389	0.054	.957	0.161	0.778	0.206	.837	−0.242	0.495	−0.490	.624

*Note*: Reference category is down‐valley for valley. Pup emergence date was fitted using orthogonal polynomials. Effects with *p* values below .05 were highlighted in bold.

## 4 DISCUSSION

We found that pup emergence date was weakly linked to female emergence date, with late‐emerging females mating in their burrow and early‐emerging females delaying reproduction. We found a positive effect of spring snowpack on the timing of pup emergence but no effect of the spring temperature. We found directional, but not stabilizing selection for pup emergence date, with pups that emerged earlier better surviving their first winter. We additionally found among‐individual variation (additive genetic + permanent environment effect) at the female level in pup emergence date, with low additive genetic variance. While there was population‐level plasticity in response to average spring snowpack, there was no inter‐individual variation in plasticity for either average spring snowpack or temperature.

We showed a weaker relationship between female and pup emergence dates than expected. Indeed, there was substantial variation in pup emergence date, with the earliest pup emerging about a month before and the latest emerging about a month later than expected based on their mother's emergence date (Figure 1). Gestation and lactation length were assumed to have a fixed duration (30 and 25 days, respectively), but there might be some among‐individual variation in both of their lengths. Yellow‐bellied marmots are considered capital breeders and mate when little to no food is available in the environment (Armitage, 2014). Therefore, the body condition of a female might shorten or lengthen gestation by a few days. During lactation, most females have emerged from their burrow, and thus both a female's body condition and micro‐environmental variation in food availability could lead to inter‐individual variation in lactation length before pups are weaned. Again, variation by a few days is expected. We also found a small effect of litter size on pup emergence date in relation to their mother emergence (Table S1). Surprisingly, one would expect that larger litters would emerge latter potentially due to the higher cost of lactation and a higher number of juveniles. However, we found a negative effect with larger litter emerging earlier.

Overall, among individual and environmental variation in gestation, lactation length, and litter size would only explain a variation of a few days in the relationship between the pup's emergence date and their mother's emergence date. Given that pups could emerge up to a month before and up to a month after expected based on their mother's emergence date, it is clear the females are in some cases delaying reproduction after emerging and, in others, able to mate in their burrow before emerging. Females delaying reproduction after emerging might be due to environmental variation, poor body conditions, and/or the absence of a male to mate with. Indeed, our results showed that pup emergence date was related to spring snowpack, with pups emerging later in springs with heavier snow (Table 1). This possibility of delaying reproduction because of the spring snowpack may also explain why pup and adult emergence dates are not similarly associated with spring temperatures. Given that the date of emergence from hibernation of adult marmots is strongly related to spring temperature (Edic et al., 2020; Inouye et al., 2000), we would also expect a positive relationship between pup emergence and spring temperatures. Yet, we find no association of spring temperature with pup emergence date (Table 1) and therefore, female marmots may delay their reproduction until there is less snow regardless of spring temperatures.

However, Andersen et al. (1976) postulated that delaying reproduction decreased fitness as the growing season was shortened for pups and females. Indeed, we found directional selection for earlier pup emergence even though females are emerging earlier from their hibernacula (Edic et al., 2020) and growing season length has increased (Cordes et al., 2020). To elaborate, litters that emerged earlier had an increased probability of surviving and increased number of pups surviving to 1‐year‐old than litters that emerged later, indicative of directional selection (Table 3). This may be a result of increased time during the growing season to forage and gain weight when pups are born earlier in the season (Monclús et al., 2014). Further, earlier‐born pups tend to be heavier at weaning than later‐born pups and this weight is positively correlated with overwinter survival (Monclús et al., 2014). We do not find a similar pattern with litter size (Table 3). Given the selective pressures for earlier births in the marmots, we would predict that females that reproduce later in the season would produce fewer but heavier pups than those females that reproduce earlier (Stearns, 1992). Indeed, it is predicted that in unfavourable environments, it is advantageous to not reproduce to your full capacity (Monclús et al., 2011; Stearns, 1992). However, in our study, it seems that regardless of the fitness costs associated with giving birth later in the season, females will give birth to the same number of pups regardless of when they emerge. Further, Monclús et al. (2014) showed that mothers did not provide more resources to pups born later in the season and thus did not reduce the fitness cost associated with the lower survival of offspring born later in the season.

Despite these existing directional selection pressures to reproduce early, the pup emergence date will show a limited or slow evolutionary response because of its low additive genetic variation (Table 2). There are two plausible explanations for this low variation. First, female marmots can re‐absorb foetuses if they are not viable. By using the pup emergence date as a proxy for the timing of reproduction, we are effectively removing all those females that may have reproduced but not given birth to any pups. This removes a potentially significant source of variation in the trait and may explain the low heritability. If females reproducing too early or late tend to reabsorb or abort their pregnancies, this may also decrease variation through stabilizing selection. Secondly, the timing of reproduction is a fitness trait, and fitness traits are generally reported to be less heritable compared to other traits (Merilä & Sheldon, 2000). This phenomenon is generally attributed to Fisher's fundamental theorem (Price & Schluter, 1991), which proposes that there should be strong selection on fitness traits that maximally increase fitness, thus resulting in lower genetic variance for fitness (Merilä & Sheldon, 2000). This may explain the pattern we observe here. There may have been strong selection on the timing of pup emergence date to increase fitness, causing a reduction in the amount of additive genetic variance present and as a result lowering the heritability of the trait. However, there have been challenges to this theorem, with suggestions that the lower heritability of fitness traits is not due to decreased additive genetic variance but rather increased residual (Merilä & Sheldon, 2000) or environmental variance (Price & Schluter, 1991). In our model, we report both low additive genetic variance and high residual variance. In addition, low heritability of fitness is not necessarily associated with slow evolution and might be a poor indicator of the rate of adaptive evolution (Bonnet et al., 2022; Hendry et al., 2018; Snyder & Ellner, 2018). We may also be lacking the statistical power necessary to detect additive genetic variance in the trait. This may be explored further as more observations are collected and more individuals are added to the pedigree.

Past selective pressures may also explain why we found no inter‐individual variation in the plasticity of pup emergence dates with spring snowpack (Figure 3). Female marmots are responding in the same way to the same changes in the average spring snowpack. Inter‐individual differences in the intercept in our plasticity model indicated that in the average environment, individuals are reproducing at different times (Nussey et al., 2007). No covariation between the intercept and the slope indicated that there is no relationship between the timing of reproduction in the average environment and how plastic an individual is (Brommer, 2013). The lack of inter‐individual variation in the slope in our population indicated that individuals do not differ in their response to changes in the environment. This may be explained by canalization (Stearns, 1982). Marmots are heavily constrained by their climate and have a relatively short period of time to reproduce and gain mass again prior to hibernating. Since there is strong selection to reproduce within a short window of time where fitness is optimized and strong selection is expected to decrease the magnitude of inter‐individual differences (Westneat et al., 2009), this may explain the lack of IxE in our study population. Predation might also drive the small variation. If females varied substantially in the timing of their reproduction in response to the same environmental conditions, pups would emerge at different times, exposing them to increased predation risk as there are fewer pups available at any given time as prey (Michel et al., 2020). We may also be lacking the statistical power necessary to detect individual variations in plasticity.

Significant sources of variation in our animal model were the valley, permanent environment, and year (Tables 1 and 2). The pup emergence date is earlier in the down valley compared to the up valley. This is to be expected as these two sites differ in elevation by about 200 m, causing a 2‐week delay in the phenology of the up‐valley compared to the down‐valley (Monclús et al., 2014). Inter‐individual variation in pup emergence date may be due to microenvironmental differences experienced by females such as burrow quality, foraging ability, or differences in environmental conditions experienced (e.g., trees preventing snow melt; van Vuren & Armitage, 1991). Inter‐annual variation in pup emergence date may be expected due to yearly variations in environmental conditions such as variation in the number of males present or amount of snow in the area. We find no association between colony and the date of pup emergence, but this may be because the permanent environment effect and colony are correlated, as females do not generally leave once they are reproductively mature (Edic et al., 2020). Colony effects that may have been confounded with the permanent environment may be the number of individuals present, as marmots can produce more pups when there are fewer individuals in the colony (Maldonado‐Chaparro et al., 2015), the number of males present in the colony, or the degree of reproductive suppression present in the colony. These factors could all impact the timing of reproduction in a colony‐specific way.

For the model examining the annual number of pups surviving their first winter, we found that more pups survive their first winter in the up‐valley compared to the down‐valley (Table 3). This is likely due to differing predation rates between the valleys, with higher predation in the down‐valley compared to the up‐valley. Predation and winter conditions are the main causes of death in marmots, and young marmots are very susceptible to predation (Armitage, 2014). For the models on litter size and the annual number of pups surviving, we report a positive effect of a mother's mass in June (Table 3). June body mass of a mother has been reported to have a positive effect on the mass of her offspring, and heavier offspring are expected to have higher chances of overwinter survival (Monclús et al., 2014). Additionally, as marmots are capital breeders, higher body masses are often associated with more resources available for reproduction, potentially explaining larger litter sizes for larger females.

There are some limitations to our dataset that may have impacted our results. First, despite our best efforts, we might have some errors on the emergence date for pups and mothers because we rely on visual observations to determine emergence. While our observation effort is high in this study, with colonies observed on a near‐daily basis and approximately 1000 h of observations logged per year, exact emergence dates may still be missed. We additionally tried to control for this by limiting our analyses to only the main colonies, as these are observed with a higher frequency than satellite colonies. Therefore, we are less likely to have missed emergence dates in the main colonies compared to the satellite colonies. Further, we are only able to use the pup emergence date as our proxy for the timing of reproduction. Being able to see inside burrows and know exactly when pups are born would provide a better estimate of the timing of reproduction in addition to identifying cases where all pups died during lactation. Similarly, being able to know when a female mated would also provide more information about pregnancy interruptions (reabsorption and abortions). Additionally, we unfortunately only have data on female emergence dates between 2003 and 2017. It would have been interesting to analyse pup and female emergence dates for more years to increase the power of our analysis. Finally, since we only have one weather station on site, the climate variables used are the same between valleys. In the future, it would be interesting to separate weather variables between the valleys since the up‐valley environment is harsher and there is a phenology delay of about 2 weeks between the valleys.

## 5 CONCLUSIONS

Overall, we report that while marmots are plastically adjusting the timing of pup emergence dates in response to changing spring snowpacks, individuals do not differ in their plasticity level. Further, pup emergence dates have low heritability, but there is selection for pups to emerge earlier. This indicates that the pup emergence date may not have an optimum time, and it is just better to emerge earlier. Without having inter‐individual variation in plasticity and without being able to evolve in response to natural selection, this population may be limited in its ability to track optimal environmental conditions for reproduction. If the climate continues to change, this may prove problematic. For instance, the length of the active season may change, altering the timing of food availability. If pups do not emerge early enough, they may not be able to gain enough mass prior to hibernation. Similarly, if the mother reproduces too late in the season, she will also be limited in her ability to gain sufficient mass for hibernation. This potential mismatch in the length of the active season and when pups emerge may impact population fitness, causing a decrease in pup and dam survival. Additionally, future research should investigate the discrepancy we report between female and pup emergence to determine the ecology behind this pattern.

## AUTHOR CONTRIBUTIONS

**Sophia St. Lawrence:** Conceptualization (equal); data curation (equal); formal analysis (lead); investigation (lead); methodology (lead); visualization (lead); writing – original draft (lead); writing – review and editing (equal). **Daniel T. Blumstein:** Funding acquisition (equal); project administration (equal); writing – review and editing (equal). **Julien G. A. Martin:** Conceptualization (supporting); data curation (lead); formal analysis (supporting); funding acquisition (equal); investigation (equal); methodology (supporting); project administration (equal); resources (lead); supervision (lead); visualization (supporting); writing – original draft (supporting); writing – review and editing (equal).

## FUNDING INFORMATION

JGAM was supported by a University of Ottawa grant and NSERC discovery grant (DGECR‐2019‐00289, RGPIN‐2019‐05000). DTB was supported by the National Geographic Society, UCLA (Faculty Senate and the Division of Life Sciences), a Rocky Mountain Biological Laboratory research fellowship, and by the National Science Foundation (I.D.B.R.‐0754247, and D.E.B.‐1119660 and 1557130 to DTB, and D.B.I. 0242960, 0731346, 1226713, and 1755522 to Rocky Mountain Biological Laboratory).

## CONFLICT OF INTEREST STATEMENT

The authors declare no conflict of interest.

### OPEN RESEARCH BADGES

This article has earned an Open Data badge for making publicly available the digitally‐shareable data necessary to reproduce the reported results. The data is available at http://doi.org/10.17605/OSF.IO/PZ4H2.

## Supporting information


Appendix S1

## Data Availability

All data and code used for statistical analysis and plots are provided via the Open Science Framework at http://doi.org/10.17605/OSF.IO/PZ4H2 (St. Lawrence, Blumstein, et al., 2022).

## References

[ece310780-bib-0001] Andersen, D. C. , Armitage, K. B. , & Hoffmann, R. S. (1976). Socioecology of marmots: Female reproductive strategies. Ecology, 57, 552–560. 10.2307/1936439

[ece310780-bib-0002] Armitage, K. B. (2014). Marmot biology: Sociality, individual fitness, and population dynamics. Cambridge University Press. 10.1017/CBO9781107284272 22017671

[ece310780-bib-0003] Auguie, B. (2017). gridExtra: Miscellaneous functions for “Grid” graphics . https://CRAN.R‐project.org/package=gridExtra

[ece310780-bib-0004] Bailey, L. D. , & van de Pol, M. (2016). Climwin: An R toolbox for climate window analysis. PLoS One, 11, e0167980. 10.1371/journal.pone.0167980 27973534 PMC5156382

[ece310780-bib-0005] Bailey, L. D. , van de Pol, M. , Adriaensen, F. , Arct, A. , Barba, E. , Bellamy, P. E. , Bonamour, S. , Bouvier, J.‐C. , Burgess, M. D. , Charmantier, A. , Cusimano, C. , Doligez, B. , Drobniak, S. M. , Dubiec, A. , Eens, M. , Eeva, T. , Ferns, P. N. , Goodenough, A. E. , Hartley, I. R. , … Visser, M. E. (2022). Bird populations most exposed to climate change are less sensitive to climatic variation. Nature Communications, 13(1), 2112. 10.1038/s41467-022-29635-4 PMC9018789 35440555

[ece310780-bib-0006] Bates, D. , Mächler, M. , Bolker, B. , & Walker, S. (2015). Fitting linear mixed‐effects models using lme4. Journal of Statistical Software, 67, 1–48. 10.18637/jss.v067.i01

[ece310780-bib-0007] Blumstein, D. T. , Lea, A. J. , Olson, L. E. , & Martin, J. G. A. (2010). Heritability of anti‐predatory traits: Vigilance and locomotor performance in marmots. Journal of Evolutionary Biology, 23, 879–887. 10.1111/j.1420-9101.2010.01967.x 20298440

[ece310780-bib-0008] Bonnet, T. , Morrissey, M. B. , de Villemereuil, P. , Alberts, S. C. , Arcese, P. , Bailey, L. D. , Boutin, S. , Brekke, P. , Brent, L. J. N. , Camenisch, G. , Charmantier, A. , Clutton‐Brock, T. H. , Cockburn, A. , Coltman, D. W. , Courtiol, A. , Davidian, E. , Evans, S. R. , Ewen, J. G. , Festa‐Bianchet, M. , … Kruuk, L. E. B. (2022). Genetic variance in fitness indicates rapid contemporary adaptive evolution in wild animals. Science, 376(6596), 1012–1016. 10.1126/science.abk0853 35617403

[ece310780-bib-0009] Boutin, S. , & Lane, J. E. (2014). Climate change and mammals: Evolutionary versus plastic responses. Evolutionary Applications, 7, 29–41. 10.1111/eva.12121 24454546 PMC3894896

[ece310780-bib-0010] Braendle, C. , Heyland, A. , & Flatt, T. (2011). Integrating mechanistiv and evolutionary analysis of life history variation. In T. Flatt & A. Heyland (Eds.), Mechanisms of life history evolution: The genetics and physiology of life history traits and trade‐offs (pp. 3–10). Oxford University Press.

[ece310780-bib-0011] Bright Ross, J. G. , Newman, C. , Buesching, C. D. , & Macdonald, D. W. (2020). What lies beneath? Population dynamics conceal pace‐of‐life and sex ratio variation, with implications for resilience to environmental change. Global Change Biology, 26, 3307–3324. 10.1111/gcb.15106 32243650

[ece310780-bib-0012] Brommer, J. E. (2000). The evolution of fitness in life‐history theory. Biological Reviews, 75, 377–404. 11034016 10.1017/s000632310000551x

[ece310780-bib-0013] Brommer, J. E. (2013). Phenotypic plasticity of labile traits in the wild. Current Zoology, 59, 485–505. 10.1093/czoolo/59.4.485

[ece310780-bib-0014] Bronson, F. H. (2009). Climate change and seasonal reproduction in mammals. Philosophical Transactions of the Royal Society B: Biological Sciences, 364, 3331–3340. 10.1098/rstb.2009.0140 PMC2781850 19833645

[ece310780-bib-0015] Butler, D. (2021). asreml: Fits the linear mixed model . R package version 4.1.0.160. www.vsni.co.uk

[ece310780-bib-0016] Charmantier, A. , McCleery, R. H. , Cole, L. R. , Perrins, C. , Kruuk, L. E. B. , & Sheldon, B. C. (2008). Adaptive phenotypic plasticity in response to climate change in a wild bird population. Science, 320, 800–803. 10.1126/science.1157174 18467590

[ece310780-bib-0017] Childs, D. Z. , Metcalf, C. J. E. , & Rees, M. (2010). Evolutionary bet‐hedging in the real world: Empirical evidence and challenges revealed by plants. Proceedings of the Royal Society B: Biological Sciences, 277, 3055–3064. 10.1098/rspb.2010.0707 PMC2982066 20573624

[ece310780-bib-0018] Cordes, L. S. , Blumstein, D. T. , Armitage, K. B. , CaraDonna, P. J. , Childs, D. Z. , Gerber, B. D. , Martin, J. G. A. , Oli, M. K. , & Ozgul, A. (2020). Contrasting effects of climate change on seasonal survival of a hibernating mammal. Proceedings of the National Academy of Sciences of the United States of America, 117, 18119–18126. 10.1073/pnas.1918584117 32631981 PMC7395557

[ece310780-bib-0019] de Villemereuil, P. , Charmantier, A. , Arlt, D. , Bize, P. , Brekke, P. , Brouwer, L. , Cockburn, A. , Côté, S. D. , Dobson, F. S. , Evans, S. R. , Festa‐Bianchet, M. , Gamelon, M. , Hamel, S. , Hegelbach, J. , Jerstad, K. , Kempenaers, B. , Kruuk, L. E. B. , Kumpula, J. , Kvalnes, T. , … Chevin, L.‐M. (2020). Fluctuating optimum and temporally variable selection on breeding date in birds and mammals. Proceedings of the National Academy of Sciences of the United States of America, 117, 31969–31978. 10.1073/pnas.2009003117 33257553 PMC7116484

[ece310780-bib-0020] Edic, M. N. , Martin, J. G. A. , & Blumstein, D. T. (2020). Heritable variation in the timing of emergence from hibernation. Evolutionary Ecology, 34, 763–776. 10.1007/s10682-020-10060-2

[ece310780-bib-0021] Fox, J. , & Weisberg, S. (2019). An R companion to applied regression (3rd ed.). Sage Publications. https://socialsciences.mcmaster.ca/jfox/Books/Companion/

[ece310780-bib-0022] Gienapp, P. , & Brommer, J. E. (2014). Evolutionary dynamics in response to climate change. In A. Charmantier , D. Garant , & L. E. B. Kruuk (Eds.), Quantitative genetics in the wild (Chapter 15, pp. 254–274). Oxford University Press.

[ece310780-bib-0023] Gienapp, P. , Reed, T. E. , & Visser, M. E. (2014). Why climate change will invariably alter selection pressures on phenology. Proceedings of the Royal Society B: Biological Sciences, 281(1793), 20141611. 10.1098/rspb.2014.1611 PMC4173688 25165771

[ece310780-bib-0024] Gienapp, P. , Teplitsky, C. , Alho, J. S. , Mills, A. , & Merilä, J. (2007). Climate change and evolution: Disentangling environmental and genetic responses. Molecular Ecology, 17, 167–178. 10.1111/j.1365-294X.2007.03413.x 18173499

[ece310780-bib-0025] Grolemund, G. , & Wickham, H. (2011). Dates and times made easy with lubridate. Journal of Statistical Software, 40, 1–25. 10.18637/jss.v040.i03

[ece310780-bib-0026] Hendry, A. P. , Schoen, D. J. , Wolak, M. E. , & Reid, J. M. (2018). The contemporary evolution of fitness. Annual Review of Ecology, Evolution, and Systematics, 49(1), 457–476.

[ece310780-bib-0027] Inouye, D. W. , Barr, B. , Armitage, K. B. , & Inouye, B. D. (2000). Climate change is affecting altitudinal migrants and hibernating species. Proceedings of the National Academy of Sciences of the United States of America, 97, 1630–1633. 10.1073/pnas.97.4.1630 10677510 PMC26486

[ece310780-bib-0028] Kroeger, S. B. , Blumstein, D. T. , Armitage, K. B. , Reid, J. M. , & Martin, J. G. A. (2018). Age, state, environment, and season dependence of senescence in body mass. Ecology and Evolution, 8, 2050–2061. 10.1002/ece3.3787 29468024 PMC5817150

[ece310780-bib-0061] Kruuk, L. E. B. , & Hadfield, J. D. (2007). How to separate genetic and environmental causes of similarity between relatives. Journal of Evolutionary Biology, 20(5), 1890–1903. Portico. 10.1111/j.1420-9101.2007.01377.x 17714306

[ece310780-bib-0029] Kuznetsova, A. , Brockhoff, P. B. , & Christensen, R. H. B. (2017). lmerTest package: Tests in linear mixed effects models. Journal of Statistical Software, 82, 1–26. 10.18637/jss.v082.i13

[ece310780-bib-0031] Lüdecke, D. (2018). Ggeffects: Tidy data frames of marginal effects from regression models. Journal of Open Source Software, 3, 772. 10.21105/joss.00772

[ece310780-bib-0032] Maldonado‐Chaparro, A. A. , Martin, J. G. A. , Armitage, K. B. , Oli, M. K. , & Blumstein, D. T. (2015). Environmentally induced phenotypic variation in wild yellow‐bellied marmots. Journal of Mammalogy, 96, 269–278. 10.1093/jmammal/gyu006

[ece310780-bib-0033] Merilä, J. , & Hendry, A. P. (2014). Climate change, adaptation, and phenotypic plasticity: The problem and the evidence. Evolutionary Applications, 7(1), 1–14. 24454544 10.1111/eva.12137 PMC3894893

[ece310780-bib-0034] Merilä, J. , & Sheldon, B. C. (2000). Lifetime reproductive success and heritability in nature. The American Naturalist, 155, 301–310. 10.1086/303330 10718727

[ece310780-bib-0035] Michel, E. S. , Strickland, B. K. , Demarais, S. , Belant, J. L. , Kautz, T. M. , Duquette, J. F. , Beyer, D. E. , Chamberlain, M. J. , Miller, K. V. , Shuman, R. M. , Kilgo, J. C. , Diefenbach, D. R. , Wallingford, B. D. , Vreeland, J. K. , Ditchkoff, S. S. , DePerno, C. S. , Moorman, C. E. , Chitwood, M. C. , & Lashely, M. A. (2020). Relative reproductive phenology and synchrony affect neonate survival in a nonprecocial ungulate. Functional Ecology, 34, 2536–2547. 10.1111/1365-2435.13680

[ece310780-bib-0036] Mills, L. S. , Zimova, M. , Oyler, J. , Running, S. , Abatzoglou, J. T. , & Lukacs, P. M. (2013). Camouflage mismatch in seasonal coat color due to decreased snow duration. Proceedings of the National Academy of Sciences of the United States of America, 110, 7360–7365. 10.1073/pnas.1222724110 23589881 PMC3645584

[ece310780-bib-0037] Monclús, R. , Pang, B. , & Blumstein, D. T. (2014). Yellow‐bellied marmots do not compensate for a late start: The role of maternal allocation in shaping life‐history trajectories. Evolutionary Ecology, 28, 721–733. 10.1007/s10682-014-9705-z

[ece310780-bib-0038] Monclús, R. , Tiumlin, J. , & Blumstein, D. T. (2011). Older mothers follow conservative strategies under predator pressure: The adaptive role of maternal glucocorticoids in yellow‐bellied marmots. Hormones and Behavior, 60, 660–665. 10.1016/j.yhbeh.2011.08.019 21930131

[ece310780-bib-0039] Nussey, D. H. , Postma, E. , Gienapp, P. , & Visser, M. E. (2005). Selection on heritable phenotypic plasticity in a wild bird population. Science, 310, 304–306. 10.1126/science.1117004 16224020

[ece310780-bib-0040] Nussey, D. H. , Wilson, A. J. , & Brommer, J. E. (2007). The evolutionary ecology of individual phenotypic plasticity in wild populations. Journal of Evolutionary Biology, 20, 831–844. 10.1111/j.1420-9101.2007.01300.x 17465894

[ece310780-bib-0041] Ozgul, A. , Childs, D. Z. , Oli, M. K. , Armitage, K. B. , Blumstein, D. T. , Olson, L. E. , Tuljapurkar, S. , & Coulson, T. (2010). Coupled dynamics of body mass and population growth in response to environmental change. Nature, 466, 482–485. 10.1038/nature09210 20651690 PMC5677226

[ece310780-bib-0042] Parmesan, C. (2006). Ecological and evolutionary responses to recent climate change. Annual Review of Ecology, Evolution, and Systematics, 37(1), 637–669.

[ece310780-bib-0043] Phillimore, A. B. , Hadfield, J. D. , Jones, O. R. , & Smithers, R. J. (2010). Differences in spawning date between populations of common frog reveal local adaptation. Proceedings of the National Academy of Sciences of the United States of America, 107, 8292–8297. 10.1073/pnas.0913792107 20404185 PMC2889515

[ece310780-bib-0044] Price, T. , & Schluter, D. (1991). On the low heritability of life‐history traits. Evolution, 45, 853–861. 10.1111/j.1558-5646.1991.tb04354.x 28564058

[ece310780-bib-0045] R Core Team . (2020). R: A language and environment for statistical computing. R Foundation for Statistical Computing. https://www.R‐project.org/

[ece310780-bib-0046] Radchuk, V. , Reed, T. , Teplitsky, C. , van de Pol, M. , Charmantier, A. , Hassall, C. , Adamík, P. , Adriaensen, F. , Ahola, M. P. , Arcese, P. , Miguel Avilés, J. , Balbontin, J. , Berg, K. S. , Borras, A. , Burthe, S. , Clobert, J. , Dehnhard, N. , de Lope, F. , Dhondt, A. A. , … Kramer‐Schadt, S. (2019). Adaptive responses of animals to climate change are most likely insufficient. Nature Communications, 10(1), 3109. 10.1038/s41467-019-10924-4 PMC6650445 31337752

[ece310780-bib-0048] Snyder, R. E. , & Ellner, S. P. (2018). Pluck or luck: Does trait variation or chance drive variation in lifetime reproductive success? The American Naturalist, 191(4), E90–E107. 10.1086/696125 29570408

[ece310780-bib-0049] St. Lawrence, S. , Blumstein, D. T. , & Martin, J. G. A. (2022). Dataset: The timing of reproduction is responding plastically, not genetically, to climate change in yellow‐bellied marmots (*Marmota flaviventer*). Archived at OSF. 10.17605/OSF.IO/PZ4H2 PMC10701086 38077518

[ece310780-bib-0050] St. Lawrence, S. , Dumas, M. N. , Petelle, M. , Blumstein, D. T. , & Martin, J. G. A. (2022). Sex‐specific reproductive strategies in wild yellow‐bellied marmots (*Marmota flaviventer*): Senescence and genetic variance in annual reproductive success differ between the sexes. Behavioral Ecology and Sociobiology, 76, 84. 10.1007/s00265-022-03191-9

[ece310780-bib-0051] Stearns, S. C. (1982). The role of development in the evolution of life histories. In J. T. Bonner (Ed.), Evolution and development (pp. 237–258). Springer. 10.1007/978-3-642-45532-2_12

[ece310780-bib-0052] Stearns, S. C. (1992). The evolution of life histories. Oxford University Press.

[ece310780-bib-0053] Svendsen, G. E. (1974). Behavioral and environmental factors in the spatial distribution and population dynamics of a yellow‐bellied marmot population. Ecology, 55, 760–771. 10.2307/1934412

[ece310780-bib-0054] van de Pol, M. , Bailey, L. D. , McLean, N. , Rijsdijk, L. , Lawson, C. R. , & Brouwer, L. (2016). Identifying the best climatic predictors in ecology and evolution. Methods in Ecology and Evolution, 7, 1246–1257. 10.1111/2041-210X.12590

[ece310780-bib-0055] van Vuren, D. , & Armitage, K. B. (1991). Duration of snow cover and its influence on life‐history variation in yellow‐bellied marmots. Canadian Journal of Zoology, 69, 1755–1758. 10.1139/z91-244

[ece310780-bib-0056] Visser, M. E. (2008). Keeping up with a warming world; assessing the rate of adaptation to climate change. Proceedings of the Royal Society B: Biological Sciences, 275, 649–659. 10.1098/rspb.2007.0997 PMC2409451 18211875

[ece310780-bib-0057] Westneat, D. F. , Stewart, I. R. K. , & Hatch, M. I. (2009). Complex interactions among temporal variables affect the plasticity of clutch size in a multi‐brooded bird. Ecology, 90, 1162–1174. 10.1890/08-0698.1 19537538

[ece310780-bib-0058] Wickham, H. (2016). ggplot2: Elegant graphics for data analysis. Springer‐Verlag. https://ggplot2.tidyverse.org

[ece310780-bib-0059] Wickham, H. , Averick, M. , Bryan, J. , Chang, W. , D'Agostino McGowan, L. , François, R. , Grolemund, G. , Hayes, A. , Henry, L. , Hester, J. , Kuhn, M. , LinPedersen, T. , Miller, E. , Bache, S. M. , Müller, K. , Ooms, J. , Robinson, D. , Seidel, D. P. , Spinu, V. , … Yutani, H. (2019). Welcome to the tidyverse. Journal of Open Source Software, 4, 1686. 10.21105/joss.01686

[ece310780-bib-0060] Wolak, M. E. (2012). nadiv: An R package to create relatedness matrices for estimating non‐additive genetic variances in animal models. Methods in Ecology and Evolution, 3, 792–796. 10.1111/j.2041-210X.2012.00213.x

