# Supplementary material for: The timing of reproduction is responding plastically, not genetically, to climate change in yellow‐bellied marmots (Marmota flaviventer)
Source: Ecol Evol. 2023 Dec 6;13(12):e10780. doi: 10.1002/ece3.10780 (PMC10701086; doi:10.1002/ece3.10780)
Supplement: Supplementary file 1 — Appendix S1 [file ECE3-13-e10780-s001.docx]

**Supplemental:**

Table S1. Linear mixed model (LMM) to analyze the association of female emergence date with pup emergence date. Pup emergence date was scaled and mean-centered. (number of females = 88, number of litters = 171).

|  | Estimate | Standard Error | DF | t-value | P-value |
| --- | --- | --- | --- | --- | --- |
| Intercept | 0.39 | 0.2 | 77.455 | 1.953 | 0.054 |
| **Female Emergence Date** | **0.282** | **0.071** | **135.936** | **3.958** | **<0.001** |
| **Litter size** | **-0.082** | **0.035** | **166.827** | **-2.335** | **0.021** |

Table S2. Summary statistics for the pruned pedigree used for the animal models presented in this paper.


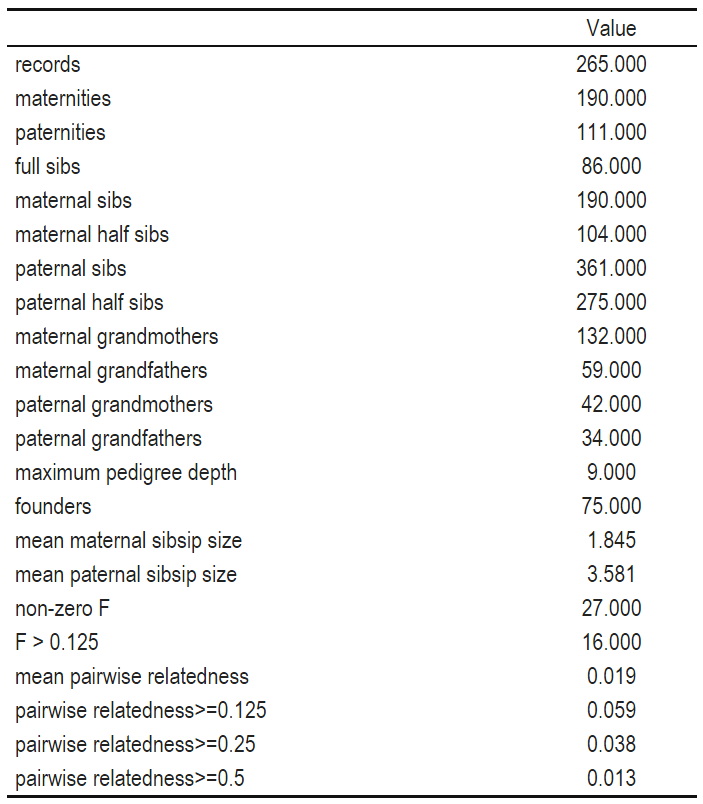


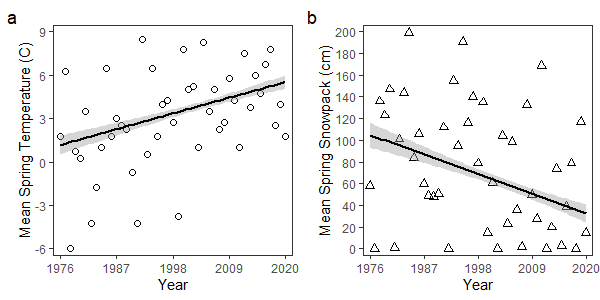


Figure S1. Relationship between year and mean spring (for definition of mean spring see results) temperature (panel a) and snowpack (panel b). Grey shading represents standard errors.


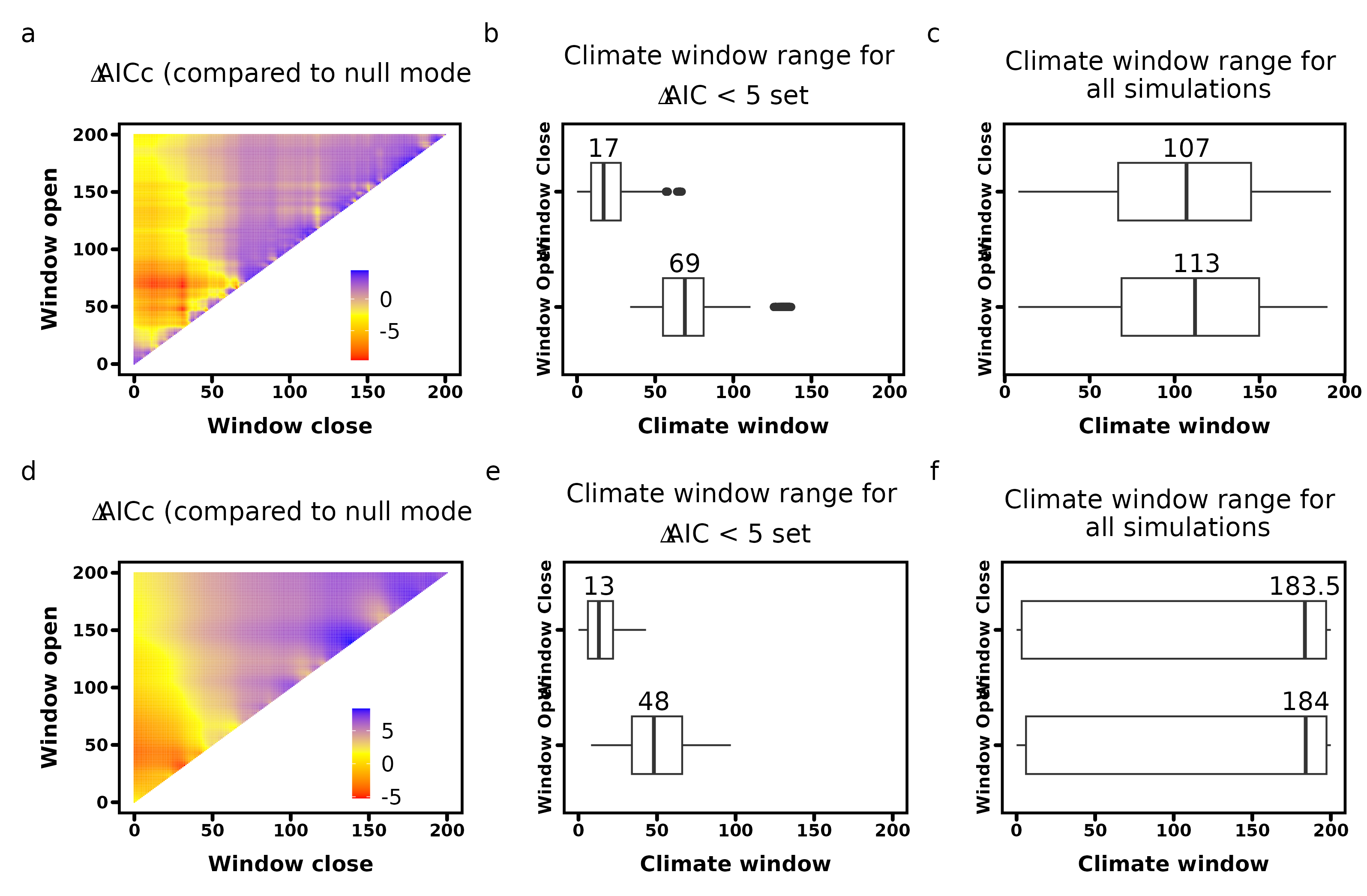
Figure S2. Output from climwin model on the association of a) average snowpack (cm) and d) average temperature (^o^C) with pup emergence date. b) and e) represent the variation in opening and closing of the windows when using all models within 5 deltaAIC. c) and f) shows the variation in opening and closing windows from best models over 500 randomisations of the data. We allowed our sliding window to vary by day starting at June 1^st^ until 200 days before. Numbers on the axes represent days before June 1^st^ with day 0 being June 1^st^ and day 200 being 200 days before June 1^st^ (November 13^th^ on average). This figure depicts the delta AICs compared between a model with year as a fixed effect and dam as a random effect and models with those effects coupled with different lengths of climate windows. The lower delta AICs, depicted in red, indicate models with the best fits.
